# Professor Dickson’s Introductory Lecture

**Published:** 1859-05

**Authors:** 


PROFESSOR DICKSON'S INTRODUCTORY LECTURE.—This beautiful and eloquent discourse was delivered at the opening of the session of the Jefferson Medical College last October, in commemoration of Dr. John K. Mitchell, whom the distinguished author had intimately known from his early youth, when they attended medical lectures in the University of Pennsylvania. The production is characterized throughout by great elegance of diction, and is a noble tribute to the memory of a man whose name, for upwards of a quarter of a century, was closely and honorably identified with the history of medical teaching and medical science in this country. Those who were so fortunate as to witness its delivery, will not soon forget the good taste, deep pathos, and stirring eloquence displayed by the speaker. Altogether, the occasion was one of the deepest interest. The discourse should be widely disseminated among the profession.

